# Understanding Childhood Neuroimmune Diseases of the Central Nervous System

**DOI:** 10.3389/fped.2019.00511

**Published:** 2019-12-19

**Authors:** Sara Matricardi, Giovanni Farello, Salvatore Savasta, Alberto Verrotti

**Affiliations:** ^1^Department of Neuropsychiatry, Children's Hospital “G. Salesi”, Ospedali Riuniti Ancona, Ancona, Italy; ^2^Pediatric Clinic, Department of Life, Health and Environmental Sciences, University of L'Aquila, L'Aquila, Italy; ^3^Department of Pediatrics, Fondazione IRCCS Policlinico San Matteo, University of Pavia, Pavia, Italy; ^4^Department of Pediatrics, University of L'Aquila, L'Aquila, Italy

**Keywords:** neuroimmune diseases, children, acquired demyelinating syndromes, autoimmune encephalitis, Rasmussen encephalitis, immunogenetic diseases, interferonopathies, hemophagocytic lymphohistiocytosis

## Abstract

Immune-mediated diseases of the central nervous system (CNS) in childhood are a heterogeneous group of rare conditions sharing the inflammatory involvement of the CNS. This review highlights the growing knowledge of childhood neuroimmune diseases that primarily affect the CNS, outlining the clinical and diagnostic features, the pathobiological mechanisms and genetics, current treatment options, and emerging challenges. The clinical spectrum of these conditions is increasingly expanded, and the underlying mechanisms of dysregulation of the immune system could vary widely. Cell-mediated and antibody-mediated disorders, infection-triggered and paraneoplastic conditions, and genetically defined mechanisms can occur in previously healthy children and can contribute to different stages of the disease. The careful evaluation of the clinical presentation and temporal course of symptoms, the specific neuroimaging and immunological findings, and the exclusion of alternative causes are mandatory in clinical practice for the syndromic diagnosis. A common feature of these conditions is that immunotherapeutic agents could modulate the clinical course and outcomes of the disease. Furthermore, specific symptomatic treatments and comprehensive multidisciplinary care are needed in the overall management. We focus on recent advances on immune-mediated demyelinating CNS disorders, autoimmune encephalitis, interferonopathies, and possible neuroimmune disorders as Rasmussen encephalitis. Better knowledge of these conditions could allow prompt diagnosis and targeted immunotherapy, to decrease morbidity and mortality as well as to improve clinical outcomes, reducing the burden of the disease due to possible long-term neuropsychiatric sequelae. Persisting controversies remain in the rigorous characterization of each specific clinical entity because of the relative rarity in children; moreover, in a large proportion of suspected neuroimmune diseases, the immune “signature” remains unidentified; treatment guidelines are mostly based on retrospective cohort studies and expert opinions; then advances in specific molecular therapies are required. In the future, a better characterization of specific immunological biomarkers may provide a useful understanding of the underlying pathobiological mechanisms of these conditions in order to individualize more tailored therapeutic options and paradigms. Multicenter collaborative research on homogeneous groups of patients who may undergo immunological studies and therapeutic trials could improve the characterization of the underlying mechanisms, the specific phenotypes, and tailored management.

## Introduction

Immune-mediated and inflammatory diseases of the central nervous system (CNS) encompass heterogeneous conditions sharing the immunological dysregulation involvement of the CNS. Specific neuroimmune diseases are rare in children, although, taken together, they are relatively common in pediatric age. The majority of these disorders have an acute onset and a self-limited course, but at other times, they can represent the first episode of a more chronic condition. Their detection and appropriate management are of paramount importance, due to the possible chronic sequelae and disease burden they could cause.

The clinical spectrum of these conditions has increasingly risen in the last years, and the underlying mechanisms of dysregulation of the immune system usually vary widely. Different pathogenic mechanisms have been identified, such as cell-mediated and antibody-mediated, infection-triggered, paraneoplastic, and genetically defined mechanisms that can occur in previously healthy children and can contribute to different stages of the disease. The diagnostic work-up comprises a careful evaluation of the clinical presentation and temporal course of symptoms, the specific neuroimaging and immunological findings, and the exclusion of alternative causes which are mandatory in clinical practice for the specific syndromic diagnosis (1).

Several possible biomarkers have also been reported to be helpful in clinical assessment and monitoring of neuroimmune disorders. Although the majority of them are non-specific, they could suggest an inflammatory or autoimmune process, and they should be considered in the overall assessment to draw diagnostic significance.

Usually, the disease course could be modulated and modified by targeted immunotherapy. Moreover, specific symptomatic treatments and prevention of secondary conditions and possible complications, as well as prolonged rehabilitation programs when required, are mandatory in the comprehensive multidisciplinary care and the overall management of these patients to improve outcomes and to reduce possible chronic sequelae.

This review highlights increasing knowledge of childhood neuroimmune diseases that primarily affect the CNS (Table 1), outlining the clinical and diagnostic features, the pathobiological mechanisms and genetics, current treatment options, and emerging challenges.

**Table 1:** Main features of childhood neuroimmune diseases of the central nervous system.

**Immune Disorder**	**Pathology**	**Clinical features**	**Neuroimaging**	**Laboratory features and/or biomarkers**	**Treatment options**
Acute disseminated encephalo-myelitis (ADEM)	• Presumed immune-cell mediated CNS disease • ADS	• Polyfocal clinical deficits • Encephalopathy • No new clinical symptoms or MRI abnormalities after 3 months from onset	• Diffuse, poorly demarcated, large (>1-2-cm) WM lesions • Rare T1-hypointense WM lesions • Deep GM lesions (e.g., basal ganglia, thalamus, or spinal cord)	• OCBs in CSF in <10% or mirror pattern • Anti-MOG-Abs in ~40%	• IV MPN • IVIg • PLEX
Multiphasic ADEM (MDEM)	• Presumed immune-cell mediated CNS disease • ADS	• Two clinical event meeting ADEM criteria, separated in time >3 months • No further events	• New or re-occurrence MRI finings • No clinically-silent new lesions between episodes	• OCBs in CSF in <10% or mirror pattern • Anti-MOG-Abs in >60%	• IV MPN • IVIg • PLEX
Clinically isolated syndrome (CIS)	• Presumed immune-cell mediated CNS disease • ADS	• Monofocal or polyfocal clinical deficits • No encephalopathy • Monophasic ON, TM, or brainstem syndrome	• T2-hyperintense and contrast-enhanced lesions in CNS area causing symptoms • No lesions elsewhere in the CNS	• None specific	• IV MPN • IVIg • PLEX
Pediatric Multiple sclerosis (MS)	• Presumed immune-cell mediated CNS disease • ADS	• At least 2 non-ADEM-like episodes • Relapsing-remitting course • Monofocal or polyfocal clinical deficits • Cognitive decline in ~30%	• DIT and DIS • T2-hyperintense, and contrast-enhanced lesions • T1- hypointense multifocal lesions (black holes) • 4 locations: periventricular, cortical or juxtacortical, infratentorial, or spinal cord	• OCBs in CSF in ~90% • (HLA)-DRB1*1501 • low serum 25(OH) vitamin D	*Acute treatment:* • IV MPN • IVIg • PLEX *DMDs:* • Interferon-β • Glatiramer acetate *(ongoing studies)* • Teriflunomide • Fingolimod • Dimethyl fumarate • Natalizumab • Ocrelizumab • Rituximab
Neuromyelitis optica spectrum disorders (NMOSDs)	• Antibody-mediated CNS disease • ADS	• Relapsing-remitting course • ON • LETM	• T2-hyperintense, T1-hypointense, and contrast-enhanced lesions • Long optic nerve • >3 contiguous spinal segments myelitis	• OCBs in CSF in ~18% • Anti-AQP4-Abs • Anti-MOG-Abs (in AQP4 negative patients)	*I line:* • IV MPN • IVIg • PLEX *II line:* • RTX • CYP *Maintenance:* • Oral prednisone • AZA • MMF
Anti-NMDAR encephalitis	• Antibody-mediated CNS disease	• Prodromal symptoms • Seizures • Movement disorders • Psychiatric behavior • Cognitive and speech dysfunction • Sleep disturbances • Decreased consciousness • Autonomic dysfunction • Relapses ~12%	• Normal • Transient non-specific T2/ FLAIR hyperintensities in multiple cortical and subcortical regions	• Anti-NMDAR Abs • Mild pleocytosis, proteinorrachia, or OCBs in CSF	*I line:* • IV MPN • IVIg • PLEX • Tumor removal (if any) *II line:* • RTX • CYP *Maintenance:* • Oral prednisone • AZA • MMF
Anti-VGKCc encephalitides	• Antibody-mediated CNS disease	• Limbic encephalitis • Seizures • Psychiatric symptoms • Movement disorders • Sleep disturbances • Morvan syndrome • Neuromyotonia • Autonomic symptoms • Relapses ~16%	• Normal • Prominent T2/FLAIR hyperintensities in mesial temporal lobes	• Anti-LGI1 Abs • Anti-Caspr2 Abs • Double-positivity • Mild pleocytosis, proteinorrachia, or OCBs in CSF	*I line:* • IV MPN • IVIg • PLEX *II line:* • RTX • CYP *Maintenance:* • Oral prednisone • AZA • MMF
Anti-GABA_A_ receptor encephalitis	• Antibody-mediated CNS disease	• Refractory seizures and status epilepticus • Cognitive and memory deficits • Movement disorders • Behavioral disturbances	• Extensive, multifocal cortical and subcortical T2/FLAIR hyperintensities	• Anti- GABA_A_ Abs • Mild pleocytosis, proteinorrachia, or OCBs in CSF	*I line:* • IV MPN • IVIg • PLEX *II line:* • RTX • CYP
					*Maintenance:* • Oral prednisone • AZA • MMF
Anti-GABA_B_ receptor encephalitis	• Antibody-mediated CNS disease	• Limbic encephalitis • Seizures • Memory deficits • Psychiatric symptoms	• Normal • T2/ FLAIR hyperintensities in mesial temporal lobes or other regions	• Anti- GABA_B_ Abs • Mild pleocytosis, proteinorrachia, or OCBs in CSF	*I line:* • IV MPN • IVIg • PLEX *II line:* • RTX • CYP *Maintenance:* • Oral prednisone • AZA • MMF
Anti-AMPAR encephalitis	• Antibody-mediated CNS disease	• Limbic encephalitis • Seizures and status epilepticus • Memory deficits • Behavioral changes	• Normal • T2/ FLAIR hyperintensities in mesial temporal lobes or other regions	• Anti-AMPAR Abs • Mild pleocytosis, proteinorrachia, or OCBs in CSF	*I line:* • IV MPN • IVIg • PLEX *II line:* • RTX • CYP *Maintenance:* • Oral prednisone • AZA • MMF
Anti-GlyR encephalitis	• Antibody-mediated CNS disease	• SPS • PERM • Limbic encephalitis • Seizures and status epilepticus • Movement disorders • Brainstem encephalitis	• Normal • Non-specific cortical and/or subcortical abnormalities	• Anti-GlyR Abs • Mild pleocytosis, proteinorrachia, or OCBs in CSF	*I line:* • IV MPN • IVIg • PLEX *II line:* • RTX • CYP *Maintenance:* • Oral prednisone • AZA • MMF
Anti-dopamine D2 receptor encephalitis	• Antibody-mediated CNS disease	• Basal Ganglia encephalitis • Lethargy • Psychiatric symptoms • Movement disorders	• Normal • T2/FLAIR hyperintensities in the basal ganglia	• Anti-dopamine D2 receptor Abs • Mild pleocytosis, proteinorrachia, or OCBs in CSF	*I line:* • IV MPN • IVIg • PLEX *II line:* • RTX • CYP *Maintenance:* • Oral prednisone • AZA • MMF
Anti-MOG associated disease	• Antibody-mediated CNS disease	• ADEM • MDEM • ON • TM • NMOSD • Non-MS course • Relapsing disease	• Long optic nerve • >3 spinal segments myelitis • T2-hyperintensities in diencephalon • Multifocal demyelination	• Anti-MOG Abs • Negative for AQP4 Abs • Typically OCBs negative in CSF	*I line:* • IV MPN • IVIg • PLEX *II line:* • RTX • CYP
					*Maintenance:* • Oral prednisone • IVIg • AZA • MMF
Autoimmune GFAP astrocytopathy	• Antibody-mediated CNS disease	• Meningo-encephalomyelitis • Seizures • Aphasia • Ataxia • Autonomic disfunction • Relapses	• Periventricular radial/patchy enhancement of diffuse subcortical hyperintensities	• Anti-GFAP Abs • Coexisting anti-NMDAR Abs	*I line:* • IV MPN • IVIg • PLEX *II line:* • RTX • CYP *Maintenance:* • Oral prednisone
					• AZA • MMF
Autoimmune encephalitides with intracellular Abs	• Immune-cell mediated CNS disease	• Seizures • Neuropsy-chological deficits • Psychiatric symptoms • Movement disorders • SPS, PERM • Limbic encephalitis • Brainstem encephalitis • Cerebellitis • Encephalo-myelitis • Opsoclonus-myoclonus	• Normal • Hyperintensity in mesial temporal lobes or other cortical and subcortical regions	• Cytotoxic T-cell mechanisms predominate • Intracellular Abs as epiphenomenon (GAD65, Hu, CRMP5, amphiphysin, Yo, Ma1, Ma2, Tr) • Tumor surveillance	*I line:* • IV MPN • IVIg • PLEX • Tumor removal (if any) *II line:* • RTX • CYP *Maintenance:* • Oral prednisone • AZA • MMF
Rasmussen encephalitis	• Acquired chronic inflammatory disease	• Refractory focal seizures • EPC • Status epilepticus • Progressive unilateral cortical deficits • Cognitive decline • Aphasia • Hemianopia	• Progressive hemispheric brain atrophy (cortex and caudate head) • T2/FLAIR hyperintensity in cortex of involved side • Ipsilateral brainstem atrophy • Contralateral cerebellar atrophy	*Brain biopsy* • T-cell encephalitis • Activated microglia • Reactive astrogliosis • Neuronal death • Unilateral brain atrophy	*Surgical:* • Hemispheric disconnection *Immunotherapy:* • Corticosteroids • IVIg • PLEX • Monoclonal antibodies • Immuno-suppressants
Aicardi Goutières syndrome	• Genetic immune-mediated CNS disease	• Progressive microcephaly • Seizures • Movement disorders • Developmental delay or regression • Chilblain skin lesions	• Cerebral calcifications • Leukodystrophy • Cerebral atrophy	• TREX1, RNASEH2A, RNASEH2B, RNASEH2C, SAMHD1, ADAR • Increased CSF levels of neopterin, biopterin, and interferons	• JAK inhibitors • Symptomatic therapies
Hemophagocytic Lymphohistiocytosis	• Genetic immune-mediated CNS disease	• Seizures • Impaired consciousness • Meningismus • Focal motor deficits • Fever • Hepatomegaly • Splenomegaly • Lymphadenopathy	• Symmetric and periventricular WM lesions (T1-hypointensity, and contrast enhancement)	• Pancytopenia • Low fibrinogen • high liver enzymes • High ferritinemia • High triglyceridemia • Hyponatremia • Pleyositosis and proteinorrachia in CSF	• Immuno-suppression • Bone-marrow transplantation

*CNS, Central Nervous System; ADS, Acquired Demyelinating Syndrome; MRI, Magnetic Resonance Imaging; WM, White Matter; GM, Gray Matterp; OCBs, Oligoclonal Bands; CSF, Cerebrospinal Fluid; Abs, Antibodies; MOG, Myelin Oligodendrocyte Glycoprotein; IV MPN, Intravenous Methylprednisolone; IVIg, Intravenous Immunoglobulins; PLEX, Plasma Exchange; DIT, Dissemination in Time; DIS, Dissemination in Space; DMDs, Disease-Modifying Drugs; ON, Optic Neuritis; TM, Transverse Myelitis; LETM, Longitudinally Extensive Transverse Myelitis; AQP4, Aquaporin-4; RXT, Rituximab; CYP, Cyclophosphamide; AZA, Azathioprine; MMF, Mycophenolate Mofetil; NMDAR, N-methyl-D-aspartate receptor; VGKCc, Voltage-gated potassium channel-complex; LGI1, Leucine-rich glioma inactivated-1; Caspr2, Contactin-associated protein-2 receptor; GABA_A_, Gamma-aminobutyric acid type A; GABA_B_, Gamma-aminobutyric acid type B; AMPAR, α-amino-3-hydroxy-5-methyl-4-isoxazolepropionic acid receptor; GlyR, Glycine receptor; SPS, Stiff-person syndrome; PERM, Progressive encephalomyelitis with rigidity and myoclonus; GFAP, Glial fibrillary acidic protein; EPC, Epilepsia partialis continua*.

## Acquired Demyelinating Syndromes

Several diseases derive from CNS dysfunction caused by immune-mediated disruption of myelin (2). Recently, the International Pediatric Multiple Sclerosis Study Group (IPMSSG) revised the criteria for pediatric acquired inflammatory demyelinating syndromes (ADSs), to better define common terminology and provide guidance in research and clinical practice (3). These syndromes included acute disseminated encephalomyelitis (ADEM), clinically isolated syndrome (CIS), neuromyelitis optica (NMO), and multiple sclerosis (MS).

The overall incidence of ADSs in pediatric age ranges from 0.6 to 1.66 per 100,000 pediatric patients per year (4–6). Across studies, the prevalence of ADSs in children may differ: 9–16% of children can present a monofocal acquired demyelinating syndrome, 19–24% ADEM, 3–22% transverse myelitis (TM), 2–36% optic neuritis (ON), and 2–4% NMO (4–12).

In pediatric age, ADEM usually occurs more frequently in children aged <10 years, while monofocal ADS such as ON and TM occur in children older than 10 years. Other disorders may be present as CIS, which could herald pediatric MS onset or remain single episodes without progression into full-blown MS. The most common CIS presentations in pediatric age are TM, ON, or brainstem, cerebellar encephalitis.

In a recent study, 23% of children were diagnosed with MS during an observational period of 4 years (4). Other studies underscore that 15–45% of children with ADSs are finally confirmed to have MS (9, 13).

Clinicians should make the proper diagnosis of the different types of ADSs, to warrant appropriate tailored management and to detect early the risk factors for a possible chronic relapsing course. Furthermore, the differential diagnosis of these entities from a broad spectrum of other white matter inflammatory diseases caused by genetic defects of the immune response, tumors, or metabolic dysfunctions is of paramount importance (2).

### Acute Disseminated Encephalomyelitis

ADEM is an inflammatory demyelinating CNS disorder characterized by encephalopathy, with a prevalence in infancy. It is a heterogeneous syndrome, rather than a specific disorder, with an incidence of 0.3–0.6 per 100,000 per year (7, 8, 14). Population-based studies report rising incidences related to the increasing distance from the equator (15). The incidence is usually higher during winter and spring months in both the United States and the United Kingdom, consistent with the link to infections.

ADEM typically presents in younger children aged 5–8 years, with male predominance (14, 16). ADEM is thought to be triggered by infections or vaccinations (16–18), with a significantly lower risk of developing ADEM post-immunization than following an infection.

ADEM is considered a monophasic disease, although the monophasic course may be confirmed after prolonged longitudinal observations over time.

In 2007, the IPMSSG proposed the criteria for pediatric ADSs to allow reliability in terminology (19). In 2013, these diagnostic criteria were revised, reflecting the most recent advances in knowledge and improving the decision-making processes (19). According to the diagnostic criteria, ADEM remains a diagnosis of exclusion of alternative causes of the demyelinating inflammatory process. The classic monophasic form usually featured as a single multifocal clinical CNS event, associated with encephalopathy (alteration of consciousness or behavioral changes not due to another systemic illness, fever, or postictal symptoms) and abnormal brain neuroimaging findings during the acute phase (within the first 3 months). Typical neuroimaging abnormalities consist of large (>1–2 cm) demyelinating lesions, usually diffuse and poorly demarcated, mainly involving the cerebral white matter. Alterations can also be detected in the deep gray matter, thalamus, basal ganglia, and spinal cord.

In a subset of patients (1–10%), a second clinical event meeting the diagnostic criteria of ADEM could occur. Multiphasic disseminated encephalomyelitis (MDEM) refers to two distinct episodes consistent with ADEM and separated by at least 3 months, but without any further subsequent events. The second clinical episode either could include new neurologic symptoms and brain lesions or may be characterized by the reoccurrence of previous ones. Relapsing episodes of subsequent ADEM occurring beyond a second encephalopathic event cannot be considered multiphasic ADEM, but a chronic relapsing demyelinating disease often fitting with a diagnosis of NMO spectrum disorders (NMOSDs) or MS. In this regard, the detection of serum anti-aquaporin-4 immunoglobulin (IgG) titer (AQP4) could suggest a diagnosis of NMOSD.

Current reports recently describe the increasing detection of myelin oligodendrocyte glycoprotein (MOG) antibodies in immune-mediated CNS disorders. MOG-antibody-positive patients are usually younger, are more frequently male, and have more often a monophasic course and a better outcome. In relapsing forms, MOG antibodies have been detected in MDEM, relapsing ON, and ADEM followed by recurrent or monophasic ON (ADEM-ON). This last clinical entity associated with anti-MOG antibodies has been described as a new relapsing phenotype, not fulfilling MS neuroimaging criteria for dissemination in space (20). The detection of anti-MOG antibodies may exclude a subsequent diagnosis of MS, although the long-term evolution of these patients needs to be further evaluated prospectively.

The clinical presentation of ADEM features an encephalopathy of acute onset associated with multiple neurologic signs and symptoms. In some cases, prodromal symptoms, such as malaise, headache, fever, drowsiness, irritability, nausea, and vomiting, may precede the abrupt onset of encephalopathy. The clinical course is rapidly progressive, with most neurologic symptoms presenting within 2–5 days (18).

The neurologic examination usually detects cranial nerve involvement, ON, slurred speech or aphasia, pyramidal signs, ataxia, and acute hemiparesis. Signs of spinal cord impairment and polyradiculoneuropathy can occur, as well as seizures, often prolonged or featuring status epilepticus. Respiratory failure due to brainstem involvement is rare. Combined central and peripheral demyelination could occur and should prompt careful differential diagnosis for other immune-mediated disorders and leukoencephalopathies of metabolic or genetic etiology.

Other clinical atypical features for ADEM that should arouse suspicion of other possible causes are persistent meningeal signs or headache, stroke-like episodes, high seizure frequency, dystonia and parkinsonism, neuropsychiatric symptoms, progressive course, history of prior developmental delay or regression, and recurrent encephalopathic events. These findings have to be differentiated from infectious or autoimmune encephalitis, genetic or metabolic disorders, or other systemic autoimmune disorders (2).

The histopathological hallmark of ADEM comprises perivascular areas of demyelination associated with inflammatory infiltrates of macrophages, T and B cells, sporadic plasma cells, and granulocytes (21). These findings differ from lesions typical of MS that consist of confluent and completely demyelinated regions associated with macrophage infiltrates mixed with reactive astrocytes. By contrast, transitional cases with both perivascular and confluent demyelination have been described in the same patient (21).

Hemorrhagic lesions have been reported in patients with acute hemorrhagic leukoencephalitis (AHL), a more aggressive and life-threatening condition. Patients with AHL may have perilesional edema and severe increased intracranial pressure.

Typical neuroimaging findings are multiple lesions that are hyperintense in T2 and fluid-attenuated inversion recovery (FLAIR) sequences, usually bilateral, asymmetric, poorly demarcated, and of different sizes that reflect pathology in myelin, as well as areas of increased diffusion coefficient values consistent with vasogenic edema. These lesions usually involve the subcortical white matter, the cortical gray and white matter junction, the thalami, the basal ganglia, the cerebellum, and the brainstem. Spinal cord involvement has also been reported, and it is characterized by large confluent lesions extending over multiple segments. Gadolinium enhancement has been reported in one-third of patients. By contrast, the presence of periventricular lesions, ovoid periventricular lesions perpendicular to the ventricular edge (Dawson fingers), lesions confined to corpus callosum or well-defined lesions, and black holes (T1 hypointense lesions) should raise suspicion for MS. Longitudinal neuroimaging reassessment with serial MRI could play a pivotal role in confirming ADEM diagnosis.

Examination of the cerebrospinal fluid (CSF) is needed to rule out treatable CNS infections, and in ADEM, it does not usually have confirmatory features. Mild pleocytosis, with a high prevalence of lymphocytes and monocytes, has been reported; mildly increased proteins may also be present. An elevated IgG index has also been reported; by contrast, oligoclonal bands (OCBs) are quite rare (<10%) or present as a mirror pattern.

The differential diagnosis of ADEM with other conditions, first of all with MS, has important therapeutic and prognostic implications. Intrathecal OCBs, a hallmark of MS, are quite rare and atypical in ADEM. Relapsing episodes should be carefully assessed, and MRI criteria, although not absolute, could help in the differential diagnostic work-up.

No randomized controlled trials are available for the management of ADEM. The treatment is usually based on observational studies and expert opinions (16, 22). In this regard, the standard of care is based on immunotherapy that contributes to faster recovery and improves outcomes. It usually comprises high-dose intravenous methylprednisolone (20–30 mg/kg/day to a maximum of 1 g/day) for 5 days, followed by steroid tapering with oral prednisone (1–2 mg/kg/day) over 4–6 weeks. Intravenous IgGs (2 g/kg, over 2–5 days) have been reported usually combined with steroids or as a second-line therapy in steroid-refractory patients. Plasma exchange (seven exchanges every day) has been recommended in refractory patients with fulminant disease.

The majority of children with ADEM have a full recovery, and brain MRI lesions often disappear as well. The neurological improvement may be detected within a few days after the start of treatment, and the recovery usually occurs within weeks rather than months. By contrast, mortality rates of 1–3% have been recently reported (23, 24), and some minor residual disability and long-term cognitive deficits have been observed in a subset of patients (25).

ADEM is an inflammatory demyelinating CNS syndrome with a strong predilection for young children. Future characterization of different possible etiologies, including CNS biomarkers as AQP4 or MOG antibodies, could lead to a better knowledge of the disease, improving differential diagnosis and management. Further, the issue of whether and how often ADEM may be the first attack of MS is still unsolved and requires multicenter collaborative research studies.

### Clinically Isolated Syndrome

CIS episodes in pediatric age are monofocal or polyfocal clinical CNS events without encephalopathy, lasting at least 24 h and due to monophasic episodes of demyelination in the absence of a previous history of CNS demyelinating disease. They include heterogeneous clinical manifestations as unilateral ON, TM, brainstem encephalitis, cerebellar tremor, ataxia, and cortical sensory or motor impairment. The longitudinal follow-up of the patient over time usually confirms or rules out the diagnosis of MS, but at the onset of an initial demyelinating CIS event, the prognosis could be less clear.

#### Pediatric Optic Neuritis

ON may manifest in almost 20–30% of children presenting a first demyelinating episode. It represents one of the most common symptoms of ADSs in pediatric age, with an incidence of 0.2 per 100,000 (8).

ON is defined as an inflammatory process involving optic nerves leading to visual defects. It is characterized by a sudden onset, with decreased visual acuity, dyschromatopsia, and deficits in the visual field. The visual loss may manifest over hours to days and rapidly increases in a few days after onset.

ON may present with optic nerve swelling and painful eye movements. Retrobulbar ON is diagnosed when typical signs and symptoms of ON are detected without optic nerve swelling.

Physical examination usually finds an afferent pupillary defect, and fundoscopic examination usually reveals optic nerve abnormalities in acute and chronic stages, as papillitis and pallor of the optic nerve.

The unilateral presentation may rapidly evolve in bilateral involvement. The majority (72%) of children <10 years old usually present with bilateral ON, while about 70% of children older than 10 years old present a unilateral clinical event.

The retinal optical coherence tomography (OCT) is useful in revealing the damage of the retinal nerve fiber layer and retinal ganglion cell body and axon layer.

Typical MRI findings are the thickening of the optic nerves on T1 sequences, T2 hyperintense signal along the optic nerve or chiasma, and gadolinium contrast enhancement. Usually, extensive bilateral lesions of the optic nerve associated with TM are detected in children positive for AQP4 featuring NMOSDs; some seronegative patients to AQP4 could carry MOG antibodies, featuring the spectrum of MOG-associated diseases.

ON in pediatric age could be highly heterogeneous and may occur as a monophasic disease or may recur isolated or associated with multifocal inflammatory conditions. Alternative etiologies as infectious, genetic, or neoplastic must be ruled out to make the proper diagnosis and tailored treatment.

ON is usually associated with functional recovery, and 77% of patients regain normal visual function within 1 year, although 34% may develop signs of a chronic inflammatory brain disease or systemic rheumatologic condition. In particular, ON is associated with a risk of a diagnosis of MS within 2 years in 13–36% of patients, and bilateral involvement usually carries a higher risk of subsequent MS than does a unilateral event. The presence of other white matter lesions outside optic nerves on MRI and the detection of OCBs in CSF at the time of diagnosis are associated with an increased risk of having MS diagnosis over time.

No clinical trials have been performed for pediatric patients with ON; thus, treatment options are mostly based on experts' clinical practice. Therapy in the pediatric population usually consists of intravenous methylprednisolone, followed by oral prednisone. A second course of high-dose steroids, intravenous IgGs, or plasma exchange could be considered in resistant patients.

Subsequent chronic immunotherapy has to be carefully evaluated according to the underlying etiology (MS/NMO/systemic rheumatologic diseases) (10).

#### Pediatric Transverse Myelitis

Pediatric TM is a demyelinating immune-mediated CNS disease and represents 20% of pediatric patients presenting a first acute event of ADS. It could be a devastating condition with variable outcomes and may be the first presenting symptom of a relapsing disease as NMO or MS.

The Transverse Myelitis Consortium Study Group defined the diagnostic criteria, which can generally be applied to the pediatric population, although they could have a lower sensory level in younger children (aged <5 years old) (11, 12).

A possible prodromal infection could be reported in the previous 30 days in up to 66% of children.

Pediatric TM usually presents with an acute onset of pain associated with sensory, motor, or autonomic symptoms due to spinal cord dysfunction. The functional impairment rapidly develops, reaching a peak after 5–6 days. During the disease course, a subset of patients may develop a persisting flaccid motor weakness. Neuroimaging assessment usually detects enhancement of spinal nerve root or cauda equina, probably due to an inflammatory disease involving both the CNS and peripheral nerve (acute TM-plus syndrome).

Other causes of myelopathy have to be ruled out, such as disorders intrinsic and extrinsic to the spinal cord. Contrast-enhanced spine imaging allows the differential diagnosis from other emergent spinal injuries, which require surgical intervention. Serum and CSF analyses allow distinguishing of specific etiologies.

Pediatric TM is usually a monophasic disorder, but previous history of episodes of myelopathy, ON, or brain lesions may suggest MS diagnosis. NMO should also be suspected with recurrent episodes of TM associated with bilateral ON, along with involvement of a spinal lesion >3 vertebral segments on MRI or the positive detection of AQP4 antibodies. The detection of MOG antibodies defines a non-MS course and may suggest a diagnosis of MOG-associated diseases; however, their role in monophasic and relapsing TM and their significance in treatment options are still unclear.

Typical MRI lesions are usually centrally located, and T2 hyperintensities involve gray and white matter. Longitudinal extensive TM is detected in most, while asymptomatic lesions may be present in almost 20–30% of children and may lead to NMO or MS evolution.

The standard empiric therapy of pediatric TM comprises high-dose intravenous methylprednisolone, but specific etiology that can worsen with steroids as infarcts and infections have to be carefully ruled out. Other treatments should be based on the underlying pathology, as cyclophosphamide in systemic rheumatologic diseases or plasma exchange in NMO. Plasma exchange or intravenous IgGs are used in affected children who do not respond to corticosteroids or those with major motor or respiratory deficits.

Pediatric patients usually present a better outcome compared with adults, with 50% reaching complete recovery by 2 years. Increased risk in mortality is associated with high cervical cord lesions determining respiratory failure.

After immunotherapy, pain rapidly improves, followed by motor dysfunctions. Sphincter disorders and sensory deficits usually require a longer time to recover, and the most common sequelae are sensory disturbances and bladder dysfunctions. One-quarter of patients may require walking aids.

The main risk factors for relapse and disability are younger age (<3 years old), female gender, longer delay of immunotherapy after symptom onset, higher spinal level involvement, presence of T1 hypointense lesions, and lack of white cells in the CSF (12).

Future collaborative research should aim to define diagnostic protocols and MRI sequences better, to delineate prognostic factors for disability and relapse, and to design clinical trials in the pediatric population.

### Neuromyelitis Optica

NMO is an ADS distinct from MS. In the pediatric population, it usually presents with bilateral optic involvement and spinal cord lesions that resemble TM.

A higher proportion of children may have a monophasic disease.

The diagnostic criteria in the pediatric population comprise ON, longitudinally extensive lesions of the spinal cord involving at least three vertebral segments, brain imaging not meeting the MS criteria, and the presence of anti-AQP4 IgG antibodies in serum (3).

The AQP4 antibodies directed against aquaporin-4 water channels are highly specific for NMO; they have pathogenic potential, and high titers in serum usually correlate with disease activity.

In 2007, the term NMOSDs was introduced and unified with the term NMO; it includes a variety of manifestations stratified according to serologic testing (NMOSD with or without anti-AQP4 antibodies) (26, 27).

Anti-AQP4 seropositive status is more often identified in female pediatric patients (3:1); it may be related to a relapsing–remitting course of the disease and could also be related with other systemic autoimmune disorders, like Sjogren syndrome, systemic lupus erythematosus (SLE), thyroid diseases, and myasthenia gravis (28).

According to the international consensus criteria, in patients diagnosed with ADEM (3), the detection of AQP4-IgG favors the diagnosis of NMOSD.

The astrocyte damage is due to complement-dependent cytotoxicity, causing leukocyte infiltration, cytokine release, and blood–brain barrier disruption, which may lead to oligodendrocyte death, myelin loss, and neuron death.

NMOSDs can also be identified in AQP4 seronegative patients, which may result in a positive status for antibodies against MOG; these patients are commonly younger at disease onset (29).

CSF analysis may show OCB-positive results in only 18% of children affected by NMO, compared with 90% of CSF samples from patients affected by MS.

In NMO, brain lesions are commonly localized in regions with high expression of the AQP4 receptors on water channels, like the optic chiasm and the area postrema of the brainstem, as well as the hypothalamus, the diencephalon, and the aqueduct. MRI lesions in the optic nerves and spinal cord are hyperintense on T2 weighted image (WI) and hypointense on T1 WI sequences with gadolinium contrast enhancement (29). Inflammatory lesions may persist for months and subsequently show an atrophic evolution (30).

Studies on OCT have demonstrated neuroaxonal damage due to damage of the retinal ganglion cells in NMO, which is higher than in MS, according to the clinical observation of more severe visual dysfunction in NMO than in MS (31).

Infections may precede disease relapses in about 30% of patients with NMO.

The treatment of acute episodes of NMO is mainly based on first-line therapies as corticosteroids and plasma exchange. In particular, plasma exchange could be of benefit in reducing AQP4 antibody titers. The treatment with intravenous IgGs has also been reported to reduce levels of antibodies. Successful treatment options comprise therapies targeting B cells such as rituximab. Other treatments as cyclophosphamide may be given in refractory cases; azathioprine, suppressing the T-cell function, may be of benefit, and mycophenolate (mofetil) has also been reported to reduce the relapse rate in NMO.

By contrast, interferon (IFN)-β used in MS may worsen NMO by increasing antibody titers; this evidence provides support that the disease activity in NMO and MS may involve different groups of T cells.

Patients who prove positive for AQP4 antibodies may be at risk for a relapsing course; therefore, long-term maintenance therapy should be taken into account even in prolonged clinical remission.

### Pediatric Multiple Sclerosis

Pediatric MS is an inflammatory demyelinating disease that in children manifests with a typical relapsing–remitting course and with lesions that are disseminated over space and time in the CNS.

This chronic condition determines focal demyelination in multiple areas of the CNS, leading to progressive neural loss and alteration in axonal integrity.

The incidence of pediatric MS ranges from 0.07 to 2.9 per 100,00 children (8).

MS onset is usually detected in youths, although in several series, MS onset in children younger than 10 years has also been reported (32–35). Nearly 3–5% of patients receive the diagnosis during childhood, and 0.3% are diagnosed before the age of 10 years (36). A higher female predominance, with an overall ratio of up to 4.5:1, is reported in patients with adolescence onset of MS; by contrast, the female-to-male ratio is lower in younger children.

Primary progressive MS is rare in children (<2%) and, if detected, should prompt the search for alternative causes such as neoplastic, mitochondrial, and neurodegenerative disorders.

Several risk factors have been associated with increased MS risk, such as female gender, onset after age of 11 years, overweight body mass index (BMI), younger age at menarche, second-hand smoking exposure, human leukocyte antigen (HLA)-DRB1^*^1501 alleles, serological evidence of remote Epstein–Barr virus (EBV) infection, and low serum level of 25(OH) vitamin D.

The lack of a pathognomonic clinical feature and diagnostic test implies that the diagnosis of MS is mostly based on the integration of the clinical, neuroimaging, and laboratory features.

Misdiagnosis represents one of the major issues in clinical practice.

In 2013, the IPMSSG (3) revised the operational criteria for pediatric MS, incorporating the 2010 McDonald criteria for MS (37). According to this definition, a diagnosis for pediatric MS fulfilled at least one of the following criteria:

1. At least two (even more) inflammatory clinical CNS events not associated with encephalopathy (non-ADEM like), separated by more than 30 days and involving more than one region of the CNS.
2. One typical MS event not associated with encephalopathy, characterized by neuroimaging features that fulfill the 2010 Revised McDonald Criteria for dissemination in space (one or more T2 hyperintense lesions in at least two of the four areas of the CNS: periventricular, cortical or juxtacortical, infratentorial, or spinal cord), and the detection at follow-up of lesion consistent with dissemination in time (the concurrent detection of gadolinium-enhanced and non-enhanced lesions or a new T2 hyperintense or gadolinium-enhanced lesion).
3. One ADEM episode followed three or more months after a CNS clinical episode not associated with encephalopathy and with new lesions on MRI consistent with the 2010 Revised McDonald criteria for dissemination in space.
4. A first, single, acute CNS episode that does not fulfill ADEM criteria and whose neuroimaging features fulfill the 2010 McDonald criteria for dissemination in space and time, applying only to patients older than 12 years.

In patients older than 11 years, the 2010 McDonald criteria are generally reliable, while in younger children with a lower risk of MS, these criteria have to be carefully evaluated. The expert panel reviewing the MS criteria agreed that the subsequent 2017 revision of the McDonald criteria should not be applied to pediatric patients at the time of one ADEM episode, but the occurrence of a further typical MS event is needed to make the diagnosis (38). The 2017 McDonald criteria are primarily applicable to adult patients experiencing a typical CIS, and they better outline all features needed to fulfill the dissemination in space and time of brain lesions and highlight the need for no better explanation for symptoms presentation. Other features that allow the diagnosis of MS comprised the detection of OCBs in patients with CIS, as well as symptomatic lesions that can be considered to demonstrate dissemination in space or in time, and cortical lesions may be used to display dissemination in space.

CSF analysis is required to rule out MS mimickers; by contrast, a normal profile does not exclude MS diagnosis. In pediatric patients, a mild pleocytosis may be detected; further, the MS hallmark of intrathecal OCBs is usually detected with a higher incidence in youths older than 11 years, and OCBs are more likely to be present in pediatric MS than in monophasic demyelinating diseases. Moreover, there is an increased likelihood of detection of OCBs and subsequent MS attacks. CSF analysis also allows us to evaluate the cytokine profiles; pro-inflammatory cytokine levels are higher in pediatric MS compared with healthy controls, and specific cytokines have been found to be linked to transcriptional regulation of T-cell subtyping and premature aging of regulatory T cells.

Given the lower incidence of MS in pediatric age, alternative differential diagnosis, as NMOSDs, could be suspected in all pediatric patients in whom a diagnosis of MS is considered. Antibody testing for anti-MOG could be helpful in patients affected by NMOSDs who prove seronegative for AQP4 antibody testing, in patients with ADEM and subsequent ON (ADEM-ON), and in those with chronic relapsing ON (20). Children with features overlapping those of ADEM, NMOSDs, and MS should be carefully evaluated.

The initial clinical manifestation of pediatric MS could be highly variable. The majority of pediatric patients have symptoms comparable to adults with a relapsing–remitting course and clinical symptoms as paresthesias, weakness, ataxia, visual loss, diplopia, or urinary dysfunction, either isolated or in combination. The first episode of pediatric MS is usually clinically monofocal, localized to a single area of CNS (39). In children younger than 10 years, MS onset is more likely to present with brainstem involvement and may also manifest polyfocal dysfunctions and encephalopathy. In these cases, the differential diagnosis with ADEM could be particularly challenging. In pediatric patients with MS, the occurrence of epileptic seizures with a variable frequency rate (5–16%) has also been reported already at disease onset. Usually, the occurrence of epileptic seizures is more common in polysymptomatic presentations and frequently associated with headache, fever, vomiting, lethargy, and altered mental status (32, 40, 41).

Compared with adult patients, children with MS may have frequent severe relapses in the first few years after disease onset. By contrast, they usually recover more fully, and the overall disease progression may be slower (42). However, the early onset of pediatric MS may lead to an increased risk of disability in early adulthood.

Risk factors for a more severe prognosis comprise a shorter interval (<1 year) between the first two attacks of MS; the incomplete recovery from a severe first attack may be an unfavorable prognostic factor, associated to an increased risk of subsequent attacks with partial recovery, hence increasing the risk of adjunctive disability over time. The involvement of the brainstem at disease onset also represents an increased risk of secondary progressive MS. Low serum level of 25(OH) vitamin D has been reported to be related to an increased risk of relapses (43).

The evolution over time of pediatric MS is usually characterized by impairment of cognitive development and mood disorders in one-third of affected children. Deficits in executive functioning, processing speed, visuomotor integration, and attention skills have been reported. Several longitudinal studies on cognitive development and neuroimaging findings highlight the correlation on neuropsychological deficits and the detection to diffusion tensor imaging (DTI) reduced fractional anisotropy in the corpus callosum and related areas of white matter, as well as reduced brain volumes, altered resting state, reduced tissue integrity, and functional connectivity (36). Lower intellectual functioning and cognitive decline after MS onset have been detected mainly in younger children in which MS may negatively affect brain growth and development of structural integrity and neural networks.

Treatment options in pediatric MS comprise the management of acute relapses, long-term immunotherapy with disease-modifying drugs, symptom management, and multidisciplinary support interventions.

Intravenous methylprednisolone is prescribed for acute relapses to shorten attacks, especially those with severe symptoms. If steroids are contraindicated or ineffective, intravenous IgGs may be used. Plasma exchange could be considered for children with severe life-threatening attacks as those involving the spinal cord or the brainstem. Acute treatment of asymptomatic new lesions on MRI is controversial, especially in children, in which steroid exposure should be minimized.

Long-term immunotherapy should be early prescribed after MS diagnosis to prevent relapses and avoid additional chronic sequelae over time. While many medications are increasingly approved for adults with MS, clinical trials on newer agents are still scant in pediatric age, and many drugs, particularly in refractory patients, are prescribed off-label. The first-line disease-modifying agents for pediatric MS are IFN-β and glatiramer acetate; currently available data provide evidence on their efficacy and safety profiles in pediatric MS (44, 45). Treatment options for pediatric patients who do not benefit from first-line therapy are less documented (44, 45). The newer agents approved for adults, as teriflunomide, fingolimod, and dimethyl fumarate have not been routinely used in pediatric patients, and clinical trials on the pediatric population are still ongoing. Higher-potency medications for MS comprise natalizumab, ocrelizumab, and rituximab, which are intravenously infused. The first two drugs are approved for adult MS, while rituximab is not Food and Drug Administration (FDA) approved and its use is still off-label in MS. Cyclophosphamide, an immunosuppressant drug, has been used in children with MS who do not respond to first-line drugs, due to its increasing experience in pediatric oncology, but newer agents show greater promise and less toxicity.

MS has heterogeneous clinical and neuroimaging features that may vary between patients and change in the same patient over time. Clinicians must be aware and have to carefully evaluate all clinical and paraclinical “red flag” findings, considering other possible causes. A wide range of conditions may be mistaken for MS (46). The increasing emphasis on timely diagnosis to reduce uncertainty and to favor early treatment with disease-modifying drugs could, by contrast, lead to an increased risk of misdiagnosis (47).

Supporting interventions for children and their families are mandatory in the comprehensive care of these patients. Social support and psychological support are mandatory to help patients and parents to cope with the impact of the MS diagnosis, potential side effects of drug therapies, and the chronic course of the disease.

Nowadays, there are no laboratory tests available to confirm the MS diagnosis, although AQP4 serological testing helps to differentiate NMOSDs from MS and testing for anti-MOG antibodies could be helpful as well, although their exact role is still controversial. In this regard, other diagnostic biomarkers have been proposed to differentiate between ADSs and to monitor CNS damage, but none of them have shown diagnostic reliability in MS, leading to this field being one of the major unmet needs and aims for future research. Emerging research is exploring the involvement of the gut microbiome, and its interaction with the cannabinoid system seems to contribute to immunological homeostasis and dysregulation (48).

Collaborative multinational networks should aim to provide advances in understanding the pathophysiologic mechanisms and to perform clinical trials in children of newer disease-modifying drugs.

## Antibody-Mediated Encephalopathies

Autoimmune encephalopathies in pediatric age comprise inflammatory brain disorders characterized by the detection of autoantibodies against cell-surface antigens as ion channels or receptors, determining reversible neuronal dysfunction. This group of encephalopathies also comprised several disorders, some of which are paraneoplastic, that are associated with autoantibodies against intracellular proteins, in which neuronal loss is common and cytotoxic T-cell mechanisms prevail. Antibodies against neuronal cell-surface antigens are deemed to be pathogenetic; by contrast, antibodies against intracellular antigens represent an epiphenomenon of the disease, although they may be considered useful biomarkers of an underlying immune-mediated process.

Autoimmune encephalitis may occur at all ages, but some disorders mainly affect children and young adults (49).

A prospective multicenter population-based study reveals that an autoimmune etiology is the third most common cause of encephalitis, after infectious and post-infectious (as ADEM) causes (50). A more recent study found that the incidence and the prevalence of autoimmune encephalitis are comparable to infectious encephalitis, and its recognition is rising over time (51). However, the exact prevalence of individual autoimmune encephalitis remains to be determined.

Autoimmune encephalopathies in pediatric age differ from the adult ones, in their clinical presentation, disease course, frequency of tumor association, prognostic factors, and treatment options.

The potential triggers of an autoimmune response against the CNS are viral encephalitis and tumors. Herpes simplex encephalitis and, likely, other viral encephalitis may favor antibodies against neuronal cell-surface proteins, causing neurological symptom relapse weeks after the onset of herpes encephalitis (52). On the other hand, some tumors may contain nerve tissues or neuronal proteins targeted by antibodies, suggesting that ectopic expression of these proteins could be involved in triggering the autoimmune response.

### Clinical Syndromes

#### Anti-*N*-methyl-d-aspartate Receptor Encephalitis

The most common form of autoimmune encephalitis in pediatric age is the anti-*N*-methyl-d-aspartate receptor (NMDAR) encephalitis that surpasses the frequency of any single viral cause of encephalitis in young patients (53).

It is a treatable disorder, characterized by acute onset of a constellation of symptoms due to widespread brain dysfunction. Initially described in 2007 as a paraneoplastic condition associated with teratoma in young women (54); nowadays, an increasing number of cases with no underlying detectable tumor have been reported mainly in children (55, 56).

To date, almost 40% of the reported patients are younger than 18 years (57, 58). The disease acute onset may be preceded by prodromal flu-like symptoms, such as fever, headache, and nausea (59). In children, presenting symptoms usually are movement disorders and epileptic seizures (60, 61), as well as insomnia and change in behavior such as irritability, temper tantrums, agitation, and regression. Adolescents usually present symptoms as in adult-onset encephalitis and mainly characterized by psychiatric symptoms as delusion and hallucinations, mood disturbances, and aggressive behavior. Typically, within the first 4 weeks of the disease, all patients, irrespective of their age, show the full-blown clinical picture characterized by at least four of the typical symptoms as regression, cognitive and memory deficits, alternating catatonia and agitation, sleep disturbances, decreased level of consciousness, dysautonomia, orofacial and limb dyskinesias, and epileptic seizures (59, 61, 62).

Approximately 5% of patients may have a monosymptomatic course (59, 60).

Clinical diagnostic criteria for definite and probable anti-NMDAR encephalitis have been recently defined in a consensus paper (63).

Brain MRI is unrevealing in almost 50% of the patients; in the remaining, transient non-specific T2 or FLAIR signal hyperintensities have been detected in multiple cortical and subcortical regions.

Interestingly, a subset of patients with anti-NMDAR encephalitis may present concurrent or separate episodes of demyelinating disorders, and by contrast, patients affected by NMOSDs or other demyelinating disorders with associated atypical symptoms, like dyskinesias or psychosis may have anti-NMDAR encephalitis (49, 57).

The electroencephalogram (EEG) findings are usually abnormal in more than 90% of these patients, with the detection of focal and diffuse slowing of the background activity and variable epileptiform abnormalities. In a subset of patients, a typical EEG pattern featuring the “extreme delta brush” may be detected (64). This pattern has been rarely identified in children, and it is usually related to a more severe and prolonged disease course.

The detection of anti-NMDAR antibodies in CSF and serum confirms the clinical diagnosis. In this regard, serum testing is less reliable, with false negatives in up to 14% of cases (65). The pathogenetic anti-NMDAR antibodies bind the NR1 subunit of the NMDAR, leading to a reversible cross-linking and internalization of the receptors, resulting in a reduction of NMDAR expression and key symptoms.

The management consists of first-line immunotherapy, including corticosteroids and intravenous IgGs, sometimes combined, and/or plasma exchange. Second-line immunotherapy, such as rituximab and cyclophosphamide, is reserved to patients non-respondent to first-line therapy. If identified, tumor must be removed (57, 59, 66). To reduce relapse rate, maintenance therapy relies on azathioprine and mycophenolate mofetil.

A favorable clinical outcome has been reported in the majority of patients (81%) (57); however, disease relapses may occur in 12–23% of cases (55). Furthermore, neuropsychological sequelae in multiple cognitive domains have been increasingly reported after remission, resulting in poor social skills and quality of life (67, 68).

#### Anti-voltage-Gated Potassium Channel Complex Encephalitides

Voltage-gated potassium channel (VGKC) complex comprises antibodies that targeted the extracellular domains of proteins complexed with the voltage-gated potassium channel, specifically leucine-rich glioma inactivated-1 (LGI1) and contactin-associated protein-2 receptor (Caspr2).

In adults, anti-LGI1 antibodies are commonly found in patients with limbic encephalitis, in a subset of whom typical faciobrachial dystonic seizures (FBDSs) may precede the onset of cognitive and psychiatric symptoms (69). Anti-Caspr2 antibodies are more commonly associated with Morvan syndrome, which is characterized by neuromyotonia, insomnia, encephalopathy, and dysautonomia (70, 71). Antibodies may cause an inhibited ligand–receptor interaction between LGI1 ADAM22/23 and reversibly reduced synaptic α-amino-3-hydroxy-5-methyl-4-isoxazolepropionate (AMPA) receptors.

These specific antibodies have been rarely reported in children, and the clinical features in this age are not completely defined. Interestingly, Schimmel et al. (72) recently described a 14-year-old boy presenting with subacute onset of memory deficits and psychiatric symptoms, left hippocampal swelling on brain MRI, and positive OCBs in CSF, associated with anti-LGI1 antibodies detected at high titers in serum (but not in CSF). He did not have faciobrachial dystonic seizures, but during the disease course, he showed paroxysmal events characterized by half-side pallor and paresthesia of the face with ptosis lasting about 1 min. The EEG recordings revealed normal findings, and he responded well to immunotherapy with good recovery. Typically, some memory disturbances and hippocampal atrophic evolution were detected.

The role of anti-VGKC complex antibodies is not well-defined in the pediatric age. High titers of these antibodies, detected by radioimmunoassay (a technique that should no longer be used in the clinical assessment), appear to be non-specific markers of neuroinflammatory disorders, and to date, they do not provide a specific syndromic diagnosis in children. In the pediatric population, many patients may be double positive, and various neurological manifestations associated with encephalopathy have been reported. Most clinical presentations are characterized by subacute cognitive and memory decline, drug-resistant seizures (none with FBDS) and/or status epilepticus, and psychiatric symptoms, as well as developmental regression, movement disorders, and insomnia. In a minority of the patients, Morvan syndrome, acquired neuromyotonia, and autonomic disturbances have been reported (73–79). Relapses may occur in 16% of the cases.

The CSF analysis usually reveals mild pleocytosis and elevated proteins. Brain MRI may detect T2/FLAIR hyperintensities in the mesial temporal lobes, with cortical and subcortical involvement. The frequency of associated tumors in children has not been defined.

Immunotherapy has been used in these pediatric cases, although often with latency from symptom onset and starting treatment. High-dose corticosteroids, intravenous IgGs, plasma exchange, and cyclophosphamide have been used with subsequent improvement in 75–100% of children. Very few reports are available on long-term outcomes, although early therapy can lead to a better recovery.

#### Anti-γ-aminobutyric Acid Type a Receptor Encephalitis

Recent reports have described anti-γ-aminobutyric acid type A (GABA_A_) receptor encephalitis in patients featuring refractory seizures and status epilepticus, as well as neuropsychiatric symptoms as cognitive and memory deficits and movement disorders. A small number of pediatric patients have been included in these reports, and their clinical presentation is characterized by a variable association of symptoms of cognitive, memory, and behavioral impairment and movement disorders, although the most common feature is a refractory/superrefractory status epilepticus (80–83).

Brain MRI usually detects extensive, multifocal cortical and subcortical T2/FLAIR hyperintensities.

The response to immunotherapy has not been defined; however, most patients may have a substantial recovery.

#### Anti-γ-aminobutyric Acid Type B Receptor Encephalitis

Anti-γ-aminobutyric acid type B (GABA_B_) receptor encephalitis presents with limbic encephalitis with early onset of seizures. Most of the clinical data are based on adult patients, in which GABA_B_ encephalitis may be usually associated with small cell lung cancer in 50% of patients.

Few cases of adolescent onset have been reported; these cases might present seizures, psychiatric symptoms, and memory deficits. Given the rarity of these antibodies in pediatric age, treatment protocols are mainly based on more common autoimmune encephalitis (84–86).

#### Anti-α-Amino-3-Hydroxy-5-Methyl-4-Isoxazolepropionic Acid Receptor Encephalitis

Anti-α-amino-3-hydroxy-5-methyl-4-isoxazolepropionic acid receptor (AMPAR) encephalitis has been mainly described in adult patients, who manifest limbic encephalitis with psychiatric symptoms and behavioral changes, memory deficits, and seizures. Most adult-onset patients are related to tumors (small cell lung cancer, thymoma, ovarian teratoma, and breast cancer).

Only two cases of pediatric-onset anti-AMPAR encephalitis have been reported, and they manifested with seizures and status epilepticus, memory deficits, and behavioral changes (87).

#### Anti-glycine Receptor Encephalitis

Anti-glycine receptor (GlyR) encephalitis has been primarily reported in adult patients, although a few cases of pediatric onset have been described (88).

The clinical presentation is usually consistent with the stiff-person syndrome (SPS) and progressive encephalomyelitis with rigidity and myoclonus (PERM), probably due to the antibody effect on the brainstem and spinal cord.

Recent reports have expanded the clinical phenotype, including limbic encephalitis, epileptic encephalopathy, brainstem encephalitis, status epilepticus, and progressive dyskinesia (89, 90).

Immunotherapy allows a marked clinical improvement.

#### Anti-dopamine D2 Receptor Encephalitis

A case series reported the presence of anti-dopamine D2 receptor (D2R) antibodies in pediatric patients featuring basal ganglia encephalitis. The clinical presentation is characterized by lethargy, psychiatric symptoms, and movement disorders, like dystonia, parkinsonism, chorea, or ataxia. In almost half of the patients, brain MRI showed T2/FLAIR hyperintensities in the basal ganglia.

These antibodies have also been detected in a small number of patients with Tourette syndrome and Sydenham chorea. Response to immunotherapy remains to be defined (91).

#### Anti-MOG-Associated Disease

Anti-MOG antibodies have been already mentioned in the “Acquired Demyelinating Syndromes” section. Indeed, these antibodies have been extensively found in children with demyelinating inflammatory disorders of the CNS, more often than adults, with high specificity (92).

Anti-MOG antibodies have been reported in about 40% of pediatric patients at first ADS, and recent studies have suggested that their detection at ADS onset may be associated with a non-MS course.

Although initially reported in monophasic syndromes, they have been increasingly identified in patients with MDEM, recurrent ON, and ADEM followed by recurrent or monophasic ON, as well as in patients with NMOSD without AQP4 antibodies.

The clinical presentation of anti-MOG antibody-associated disorders may vary with age, with ADEM-like presentation in younger children and opticospinal presentation in children older than 9 years old.

The seroconversion to anti-MOG antibodies negative status is not frequent; on the other hand, patients may prove positive even when asymptomatic. Disease relapses may occur in children who become seronegative, while relapse-free patients may have persisting high titers of antibodies (92).

These findings suggest that the antibody status should be evaluated only in conjunction with the clinical presentation and evolution to guide management. The relationship between anti-MOG antibody titers and disease activity remains to be investigated deeper.

These antibodies bound the extracellular domain of MOG antigens and also induce natural cell-mediated killing of MOG-expressing cells *in vitro*. Myelin changes and altered expression of axonal proteins usually occur, in the absence of axonal loss and astrocyte or neuronal death. In this regard, anti-MOG antibodies may be a marker for persisting demyelination (93).

Patients with NMOSD who have anti-MOG antibodies as well as anti-AQP4 have been reported to have a milder phenotype than patients without anti-MOG (94).

In a recent study, Baumann et al. (95) compared the clinical and radiological findings of pediatric patients with ADEM and with and without anti-MOG antibodies; they highlighted that the group with anti-MOG had an MRI characterized by large, bilateral, and widespread lesions in the brain, with longitudinally extensive TM and a favorable outcome compared with children without anti-MOG.

Patients with anti-MOG-associated disorders are empirically treated with corticosteroids and/or intravenous IgGs. Most of them have a favorable outcome, but a subset of patients may have a persisting disability, as a result of the first attack. Furthermore, disease relapses are commonly observed either during corticosteroids weaning or within 2 months of corticosteroid withdrawal. All these findings could suggest that a longer duration of initial treatment should be needed and that time to treatment plays a pivotal role in the prevention of permanent sequelae.

It remains unclear whether these patients should be treated as AQP4-positive patients. Disease-modifying drugs used in MS like IFN and natalizumab should worsen NMOSD (both AQP4 positive and AQP4 negative); therefore, their use has to be carefully evaluated in other antibody-mediated demyelinating diseases.

The spectrum of anti-MOG-associated disorders in pediatric age needs to be further defined, and future research studies are required to determine diagnostic and therapeutic options better.

#### Autoimmune Glial Fibrillar Acidic Protein Astrocytopathy

A new type of meningoencephalitis known as autoimmune glial fibrillary acidic protein (GFAP) astrocytopathy has been recently defined (96–99). To date, very few pediatric cases have been identified. In a prospective study, Dubey et al. (100) reported 10 pediatric patients with a clinical presentation similar to adult onset and characterized by acute meningoencephalitis with and without myelitis, refractory seizures, aphasia, autonomic dysfunction, ataxia, and, in some patients, coexisting anti-NMDAR antibodies. None of them had detectable tumors compared with 14% in adults. Most of the patients had a favorable response to immunotherapy. In this regard, the majority of the patients usually respond to first-line treatment with high-dose corticosteroids or intravenous IgGs; in a subset of the cases, more prolonged immunomodulation may be warranted with rituximab, cyclophosphamide, or mycophenolate mofetil. Refractoriness to initial first-line treatments should raise suspicion for coexisting anti-NMDAR antibodies or underlying tumor.

#### Autoimmune Encephalitides With Antibodies Targeting Intracellular Antigens

Glutamic acid decarboxylase 65 (GAD65) is an intracellular enzyme expressed in GABAergic neurons and pancreatic β-cells. Anti-GAD65 antibodies have been detected in different neurological disorders as SPS, cerebellar ataxia, autoimmune epilepsies, and limbic encephalitis. These antibodies may also coexist with other pathogenic antibodies, thus confounding the specific respective role.

Pediatric patients with high titers of anti-GAD65 in the CSF may have focal seizures (usually of temporal lobe onset), cognitive and memory deficits, developmental regression, and psychiatric symptoms. Immunotherapy may improve outcomes, although several pediatric patients may be left with persistent memory deficits and chronic epilepsy.

Other autoimmune neurological disorders due to antibodies against intracellular antigens comprised a wide range of clinical manifestations, including limbic encephalitis, brainstem encephalitis, cerebellitis, neuropathies, and diffuse encephalomyelitis. These disorders are usually paraneoplastic mainly in adult patients, and a range of tumors have been found to be associated, as small cell lung cancer (anti-Hu, CRMP5, and amphiphysin), breast cancer (CRMP5, anti-Yo, and amphiphysin), testicular teratoma (MA2), Hodgkin lymphoma (Tr antibodies), and thymoma (CRMP5).

The reported cases of pediatric onset associated with anti-Hu antibodies had neuroblastoma and opsoclonus–myoclonus (two cases) or classic non-paraneoplastic limbic encephalitis (six cases). Other paraneoplastic antibodies are very rare in the pediatric population; a single case with anti-Yo manifested subacute onset cerebellar ataxia (no tumor was found to be associated); MA2 has been identified in a 14-year-old girl with severe encephalopathy, seizures, and status dystonicus; and Tr has been detected in a 12-year-old boy with Hodgkin lymphoma.

Paraneoplastic neurological conditions, although very rare in children, should be recognized early to prompt immunotherapy alongside tumor-specific management and surveillance.

The T-cell-mediated cytotoxicity, in conjunction with the intracellular antigen location, may be responsible for the poor clinical response to immunotherapies. Pediatric patients who have experienced paraneoplastic encephalitis often have sequelae as refractory epilepsy and cognitive impairment (101, 102).

### Treatments and Outcomes

To date, current treatment options and recommendations are mainly based on retrospective studies and expert opinions. The general therapeutic approach comprises the treatment or removal of the immunologic trigger when present, such as infections or tumors, and early start of first-line immunotherapy. First-line drugs include corticosteroids, which have a broad mechanism of action on the immune system and may modulate the blood–brain barrier. Intravenous IgGs are often given combined with the corticosteroids and may have a broad immunomodulatory potential. Plasma exchange can also be applied, preferably before the intravenous IgGs. In most autoimmune encephalitis, the antibody production and inflammatory changes may occur behind the blood–brain barrier, thus leading to limited efficacy in such cases of intravenous IgGs and plasma exchange compared with other systemic antibody-mediated diseases (such as myasthenia gravis). In patients who do not respond to first-line drugs in the first 2 weeks of treatment, second-line therapy with rituximab or cyclophosphamide should be started. Rituximab shows a B-cell depletion mechanism of action, resulting in a significant reduction of pro-inflammatory CD4 and CD8 T-cell responses, underlining broad immunosuppressant properties. In this regard, it is effective in refractory cases, and it may reduce the risk of relapses. Cyclophosphamide is the alternative second-line drug, and it has a broad cellular immunosuppressive action. It should be given to refractory cases with severe disease, weighing its potential for long-term infertility and secondary malignancy (66, 103).

A subset of patients may benefit from maintenance immunotherapy. Oral prednisone or monthly intravenous IgGs may also be prescribed after clinical remission, to reduce relapse rate. When steroid dependence is proven or a relapsing course is detected in steroid-responsive patients, steroid-sparing agents as azathioprine and mycophenolate mofetil are commonly used.

When the first- and second-line treatments fail to achieve disease remission, very limited options might be available. Some recent reports suggest the use of bortezomib in severe refractory adult patients affected by anti-NMDAR encephalitis. It is a protease that inhibits pro-inflammatory signaling cascades and reduces plasma cell and antibody production (104). Tocilizumab, which targeted the pleiotropic pro-inflammatory cytokine interleukin (IL)-6, may be useful in autoimmune encephalitis as anti-NMDAR encephalitis and anti-MOG antibody-associated diseases that usually present elevated CSF IL-6 (105). Other treatments suggested in refractory cases are intrathecal steroids and methotrexate, which have been used in a few pediatric patients with anti-NMDAR encephalitis (106).

Alongside immunomodulatory therapies, symptomatic treatments should be provided. The main agents to be used are the sedative ones targeted mostly on sleep disturbances and psychiatric and behavioral disorders. Benzodiazepines, antiepileptic drugs (AEDs), and some psychotropic drugs as quetiapine are often used with some benefit (107).

The speed of recovery, the degree of neurological sequelae, and the frequency of the relapses may vary according to the type of autoimmune encephalitis. For all types of encephalitis, early immunotherapy may be associated with a favorable outcome and a more rapid recovery, and in some cases, the treatment with second-line drugs may reduce the risk of relapses.

The therapeutic management of pediatric patients must weigh the long-term implications of each intervention. In this regard, clinicians have to consider the potential effect of given immunotherapy on the developing neuroimmunological system. The use of first-line treatments is justified in the acute phase of the illness; by contrast, the long-term therapy with immunomodulatory and immunosuppressant agents must be carefully evaluated, considering the patient's age and the risk of disease relapses.

The gold standard in the decision-making process remains the clinical assessment of the child and the evaluation of all paraclinical findings. In this regard, consensus treatment recommendations should be warranted.

### Future Priorities and Challenges

In the last 10 years, an increasing number of autoantibody and associated syndromes have been described, changing the clinical practice in many pediatric neurological conditions. However, a large proportion of children with suspected immune-mediated CNS disorders do not have identifiable known antibodies, leading to many diagnostic and therapeutic challenges. Recently, clinical guidelines on diagnostic criteria for “possible” and “probable” autoimmune encephalitis encourage clinicians to start early immunotherapy while awaiting results for autoantibody testing. In this regard, the development of pediatric-centered consensus definitions for negative patients suspected for autoimmune encephalopathy is warranted. On the other hand, other antibody targets might be defined in the future.

Other key challenges remain such as the timing for second-line therapies, the disease activity monitoring, and the early recognition of patients who could have a poor response to treatments or who could be at higher risk for disease relapses. Forthcoming research studies should better define proper tools for monitoring patients with autoimmune encephalitis and neuroprotective strategies.

To date, current knowledge does not allow specific correlations between antibody titers and disease activity; in many conditions, antibodies remain detectable, usually at low titers, after clinical recovery, suggesting the need to define biomarkers that could advise prognosis and guide the treatment decision-making process. In this regard, paraclinical tools, like electroencephalography, neuropsychological testing, and advanced neuroimaging techniques, might add useful insights to the diagnostic evaluation and the assessment of treatment response.

Future studies are also warranted to better understand the underlying pathogenic role of the antibodies and the process by which they could alter the structure and function of the neuronal synaptic proteins, leading to specific clinical symptoms. A better understanding of these mechanisms could allow the development of future targeted treatment strategies.

## Rasmussen Encephalitis

Rasmussen encephalitis (RE) is a rare, acquired devastating chronic inflammatory disease, which progressively affects one brain hemisphere and typically begins during childhood.

RE is clinically characterized by refractory focal seizures, progressive worsening of unilateral neurological deficits, and cognitive decline. The neuropathology is consistent with T-cell encephalitis associated with activated microglia and reactive astrogliosis, which progressively lead to neuronal cell death and unilateral brain atrophy.

It was first described in 1958 by Theodore Rasmussen (108) in three children with the typical phenotype. Subsequently, an increasing number of reports have widened the clinical characterization, and new insights have been added to current knowledge (109, 110). However, despite increasing evidence of the underlying inflammatory process, the primary cause of RE remains to be defined.

### Clinical Features

Typical RE starts during childhood and may affect both males and females. The median age at disease onset is 5 years in previously healthy children; in almost half of the patients, febrile illness in the months before onset or a remote head trauma might be reported. Seizures or status epilepticus usually mark the disease onset; seldom, seizures may be preceded by mild progressive hemiparesis or, even more rarely, by movement disorders involving one body side like hemidystonia or hemiathetosis. In some patients, a “prodromal stage,” usually lasting 0 months to 8 years, may be characterized by infrequent seizures and mild hemiparesis. The “acute stage,” which in most patients appears to be the clinical presentation of the disease, is marked by high seizure frequency, usually of polymorphic semiology (virtually all types of focal seizures may occur, reflecting newly affected areas of inflammation in the involved hemisphere), epilepsia partialis continua (EPC), and episodes of status epilepticus (111). A rapid neurologic and cognitive deterioration is usually detected during this phase, featuring hemiparesis, cortical sensory loss, hemianopia, behavioral changes, learning disabilities, and when the dominant hemisphere is affected, aphasia. Finally, a “residual stage” with stable motor and cognitive neurological deficits and low seizure frequency is observed, although relapse of refractory seizures and further worsening of cognitive functioning may be detected.

Among less common clinical presentations, a late-onset form of adolescent–adult onset has been increasingly recognized and accounts for about 10% of patients. These cases have a milder and protracted clinical course, with focal seizures and less severe EPC, less severe motor and cognitive deterioration, less pronounced hemispheric atrophy, and better patient response to immunotherapies (112–115).

In some cases of adolescent–adult onset, the progression of brain atrophy is lower and may remain circumscribed, and then severe clinical symptoms and protracted course are detected (116). Recently, progressive brain atrophy is observed in some patients without seizures or already present at seizure onset, thus confirming that seizures do not induce the progression of the brain atrophy (117). About 10% of patients may have clinical and neuroimaging findings consistent with RE diagnosis, but in which neuropathology detected the coexistence of preexisting brain lesions, either dysplastic or neoplastic, featuring a double pathology (118, 119). Moreover, recent reports described the detection of different antibodies against cell-surface neuronal antigens (as anti-NMDAR and VGKC) and intracellular epitopes (anti-Hu) in RE patients (120–122).

### Diagnosis

The diagnosis of RE is based on clinical features and laboratory findings that support the hypothesis of unilateral progressive brain damage. The diagnosis may be particularly challenging in the initial stages before the full-blown clinical picture and MRI appear (111).

The European consensus panel proposed formal diagnostic criteria in 2005 (123) based on clinical and paraclinical (EEG, MRI, and neuropathology) findings. The clinical criteria comprise two steps. In the first is the presence of focal seizures, with or without EPC, and unilateral cortical deficits, associated with hemispheric slowing of the background activity on EEG, with or without epileptiform activity and unilateral seizure onset. Second is brain MRI featuring hemispheric focal cortical atrophy and at least one of the following: gray or white matter T2/FLAIR hyperintensity and hyperintense signal or atrophy of the ipsilateral caudate head. If these criteria are fulfilled, the brain biopsy is not needed (Part A). If the criteria are not satisfied, the progression of clinical deficits must be associated with progressive hemispheric atrophy detected on the longitudinal brain MRI or by brain biopsy showing T-cell-mediated encephalitis, with activated microglia and astrogliosis (Part B).

#### Diagnostic Tests

No laboratory tools are available to support the diagnosis of RE.

CSF analysis may show normal findings or may show a mild increase in lymphocytes and proteins. OCBs may be found in almost half of the patients (111). CSF normal or abnormal findings do not confirm or exclude the diagnosis of RE, but it should be performed to rule out alternative causes.

The detection of anti-glutamate receptor 3 (GluR3) antibodies is not a key finding of RE since they do not allow differentiation between RE cases and other epilepsies.

In the early stages, the EEG findings are usually limited to the affected hemisphere and are characterized by the unilateral focal slowing of the background activity and sleep disorganization, epileptiform abnormalities, interictal and ictal hemispheric multifocal abnormalities, and subclinical ictal discharges (111). During disease course and progression, the EEG features are characterized by an increase of the epileptiform abnormalities, which tend to spread and involve the contralateral hemisphere (124). However, seizure onset, although multifocal, remains confined to the affected hemisphere.

Progressive hemispheric brain atrophy is the hallmark of the disease; however, early neuroimaging changes may suggest the diagnosis. In this regard, mild cortical atrophy mainly involving the insular and peri-insular regions associated with ipsilateral ventricular enlargement, and cortical and subcortical T2/FLAIR hyperintensities may raise diagnostic suspicion. Furthermore, in the early stages of the disease, atrophy and T2 hyperintensity of the caudate head are frequently observed (125). During the disease course, the caudate atrophy worsened, and unilateral cortical atrophy progressively involved one hemisphere.

A recent study compared the volumetric MRIs of RE patients; in particular, the volumetric measures of hemispheric and frontal lobe ratios may support the diagnosis in early stages and may serve as a marker of disease progression (126).

Brain biopsy should be useful in atypical cases; it has to be performed in an open procedure, in non-eloquent areas, and it should include the meninges and gray and white matter to detect the multifocal changes of the T-cell encephalitis, microglia activation and nodules, reactive gliosis, and neuronal loss (127).

#### Differential Diagnosis

RE, particularly in the early stages, may be confused with other conditions characterized by unilateral or focal brain damage or EPC. It should be differentiated from other epileptic conditions resulting from focal or hemispheric dysplasia, tumor, neurocutaneous syndromes (as Sturge–Weber), stroke, or hemiconvulsion–hemiplegia–epilepsy. Other causes of EPC are neurometabolic and degenerative syndromes (128). It should also be differentiated from other inflammatory and infectious brain diseases (109).

### Etiopathogenesis

Despite increasing knowledge and clinical definition and characterization, the etiology of RE remains unknown.

Recent findings on a pivotal role of T-cell response have drawn the attention to a potential virus antigen as the triggering event, although extensive studies aimed to detect viral antigens or genetic material of viral origin gave inconsistent results (129).

Furthermore, the detection of autoantibodies in RE patients could be carefully evaluated, because it could be secondary to brain damage and because humoral immunity may not be the primary cause.

Infiltrating T cells have been characterized as CD8^+^ cells that were in close contact with major histocompatibility complex (MHC) class I-positive neurons. These cells may contain granules positive for granzyme B^+^, a protease released by activated cytotoxic T cells into target cells that undergo apoptosis. In this regard, T-cell-mediated death of astrocytes is a specific feature of RE (130). Furthermore, peripheral CD8^+^ T-cell expansion correlates with disease severity.

The presence of resident memory T cells in the parenchymal tissue of RE patients has been recently demonstrated (131), suggesting that cellular immune response may occur early and may herald clinical presentation.

### Treatments

The management of RE should include the treatment of symptoms and the underlying inflammation. Awareness of the disease increases with age, and the involvement particularly of adolescent patients in the decision-making process may be pivotal (132).

Given the different severity of the disease in different stages, the therapeutic strategies must be tailored in each patient, considering the management of seizures and the ability to cope with long-term neurological deficits as well.

AED treatment is invariability present, taking into account drug–drug interactions and side effects, mainly in polytherapy (133–135).

Surgical treatment with hemispheric disconnection is effective in improving seizures and motor and cognitive deterioration as well. Recent outcome data showed complete seizure control in 81% of cases and AED withdrawal in 76% (136, 137). Cognitive outcomes were influenced by the timing of surgery after disease onset, with better results in younger children operated soon after (136, 138).

The decision regarding indications and timing is particularly challenging in adolescent and adult patients. In selected late-onset patients, conservative options with cortical resections have been performed (139).

Immunomodulatory treatments have been increasingly used, considering their anti-inflammatory and immunomodulating properties, the regulation of the blood–brain barrier, and the antiepileptic effect. Corticosteroids and IgGs have been widely used, more recently succeeding the insight on humoral and cellular immunity; different treatment options, like plasma exchange, monoclonal antibodies, and immunosuppressants, have been proposed (140–145).

In this regard, corticosteroids, IgGs, and plasma exchange appear to be effective in acute stages on frequent seizures and status epilepticus with transient improvement, while immunomodulants and immunosuppressive drugs may be effective in slowing motor and cognitive decline, with minimal or no effect on seizure burden. Given the limited and transitory effect of immunomodulatory treatments of seizure and disease progression, they should be reserved to selected patients, to those not or not yet suitable for surgery, or in late-onset cases (110).

## Immunogenetic Diseases

### Genetic Interferonopathies

The interferonopathies comprise a group of monogenetic disorders characterized by alteration in the homeostatic control of the IFN-mediated responses. Despite a wide variety of phenotypic expression and severity, these disorders may present a considerable overlap in clinical features (145).

Interferonopathies typically present in early life and may mimic congenital infections or may be mistaken for other systemic autoimmune diseases as SLE or juvenile dermatomyositis (146).

Among interferonopathies, the Aicardi–Goutières syndrome (AGS) is an early-onset progressive brain disease that mimics the sequelae of prenatal viral infection. Increasing genetic variants have been identified over time, and other phenotypic features have also been described to be associated. AGS is linked to mutations affecting RNA metabolism or to proteins that prevent the self-activation of innate immunity by cell-intrinsic components. This brain disease presents as an early-onset encephalopathy characterized by basal ganglia calcification and abnormalities in the cerebral white matter. A subset of patients (20%) have severe neurological compromise and dysfunction identified immediately after birth, presenting with spasticity, dystonia, epileptic seizures, cortical blindness, and progressive microcephaly. Usually, this very-early-onset of AGS is associated with biallelic pathogenic variants in RNASEH2A, RNASEH2C, or TREX1. Fever of unknown origin and unidentified infective etiology may be present. Some patients may also develop thrombocytopenia, hepatosplenomegaly, and transaminitis. Chilblain-like lesions or other more severe skin manifestations may be detected. Some cases may have prominent lupus-like features, but a full-blown picture of SLE is very unusual (147).

Late-onset presentation of AGS, after a symptom-free period of some months, has also been reported. Non-specific symptoms, such as irritability, sleep disturbances, and poor feeding, and low-grade pyrexias, may mark the disease onset. These symptoms of subacute encephalopathy are usually followed by psychomotor delay or regression and secondary microcephaly. The encephalopathic phase may last some months, and after this time, the clinical picture usually stabilizes. The late-onset presentation may occur several months after initial normal development, and it is sometimes associated with preserved neurologic function. The late-onset AGS is associated with biallelic pathogenic variants in RNASEH2B, SAMHD1, or ADAR; but it could also be detected in patients with autosomal dominant heterozygous pathogenic variants in ADAR or IFIH1 (146).

Mutations in ADAR have also been reported to be associated with acute bilateral striatal necrosis. Furthermore, patients with biallelic variants in SAMHD1 may present intracerebral vasculopathy, including stenosis and aneurysms; these patients may also develop chilblains and glaucoma. Overall mortality rate is high in AGS patients, and it is usually related to genotype: 34% of cases carrying RNASEH2A, RNASEH2C, and TREX1 pathogenic variants died, the majority of whom (81%) by the age of 10 years old; otherwise, only the 8% of patients carrying RNASEH2B pathogenic variants may die (146).

Neuroimaging typically features intracerebral calcifications, leukodystrophy, and cerebral atrophy. The localization and extent of calcifications can be variable. In this regard, AGS should be considered for the differential diagnosis of other causes of leukoencephalopathy (147). CSF analysis usually reveals chronic leukocytosis, predominantly lymphocytes, elevated neopterin, and elevated IFN-α activity, which declines over time. The “IFN signature” is present in almost 100% of patients carrying pathogenic mutations in TREX1, RNASEH2A, RNASEH2C, SAMHD1, ADAR, and IFIH1 and in ~30% of patients carrying pathogenic mutations in RNASEH2B (148).

The management of AGS is generally supportive and symptomatic, with antiepileptic drugs to treat epileptic seizures (149–151) or botulinum toxin injections or levodopa to manage spasticity and dystonia. Corticosteroids have been shown to lower IFN-α concentrations in CSF and may be of benefit for skin manifestations. New strategies to block IFN signaling are based on the use of Janus kinase (JAK) inhibition, which may be of benefit in AGS as in other interferonopathies (152, 153).

### Hemophagocytic Lymphohistiocytosis

Congenital immunogenic disorders are rare, but most of them are characterized by a prevalent involvement of the CNS as the hemophagocytic lymphohistiocytosis (HLH). HLH comprises several different forms including familial HLH (FHLH), Chediak–Higashi disease, Griscelli disease, and Purtilo disease. The most frequent is FHLH, which results from genetic defects impairing the cytotoxicity of CD8 T cells (154).

These genetic defects may remain asymptomatic until a trigger, usually a viral infection, induces the uncontrolled polyclonal activation of CD8 T cells and macrophages, resulting in an overproduction of cytokines. The median age at HLH onset is usually 2.5 months, but the range can vary from 0 to 190 months (155).

Presenting symptoms may be not restricted to CNS, and fever, hepatomegaly, splenomegaly, and lymphadenopathy are commonly reported. Neurological symptoms consisted of seizures, impaired consciousness, meningismus, and focal motor deficits. Other findings included pancytopenia, low fibrinogen levels, high liver enzymes, hyperferritinemia, hypertriglyceridemia, and hyponatremia.

Key findings that may suggest the diagnosis include young age at presentation, consanguinity of parents, and early death of a sibling.

CSF analysis may be normal in almost half of the patients; in the remaining half, it may reveal high protein titer, high cellularity, and features of hemophagocytosis. Neuroimaging abnormalities comprise white matter lesions that are usually symmetric, with periventricular localization and with a hypointense signal in T1 sequences and gadolinium enhancement in some cases (156).

Early diagnosis can be based on immunological testing (including analysis of perforin expression in lymphocytes and the cytotoxic ability of the patient's CD8 cells) before the identification of pathogenic mutation. Immunosuppression followed by bone marrow transplantation is essential, while all other treatments invariably lead to relapses and necrosis of brain parenchyma (157).

## Discussion

Better knowledge of these conditions could allow prompt diagnosis and targeted immunotherapy, to decrease morbidity and mortality as well as to improve clinical outcomes, reducing the burden of the disease due to possible long-term neuropsychiatric sequelae.

Persisting controversies remain in the rigorous characterization of each specific clinical entity because of the relative rarity in children, and in a large proportion of suspected neuroimmune diseases, the immune “signature” remains unidentified; treatment guidelines are mostly based on retrospective cohort studies and expert opinions; then advances in specific molecular therapies are required.

Advances in specific CNS biomarker characterization may prove useful in the future in providing a better understanding of etiology, differential diagnosis, and tailored interventions.

In the future, the characterization of prognostic markers could be warranted in order to individualize therapeutic options and paradigms.

Tailored management could be developed based on increasing knowledge of the underlying pathobiological mechanisms of these conditions and by more collaborative clinical data recordings.

Multicenter collaborative network research on homogeneous groups of patients who may undergo immunological studies and therapeutic trials could improve the characterization of the underlying mechanisms, the specific phenotypes, and tailored management. In this regard, all these efforts could be of great impact and have a positive improvement in the quality of life of ill children and their families.

## Author Contributions

SM contributed to the conception of the review and wrote the manuscript. GF, SS, and AV contributed to the conception of the review and revised the manuscript.

### Conflict of Interest

The authors declare that the research was conducted in the absence of any commercial or financial relationships that could be construed as a potential conflict of interest.
